# Sex Difference in the Risk of Dementia in Patients with Atrial Fibrillation

**DOI:** 10.3390/diagnostics11050760

**Published:** 2021-04-23

**Authors:** Yung-Lung Chen, Joseph Chen, Hui-Ting Wang, Ya-Ting Chang, Shaur-Zheng Chong, Shukai Hsueh, Chang-Ming Chung, Yu-Sheng Lin

**Affiliations:** 1 Division of Cardiology and Department of Internal Medicine, Kaohsiung Chang Gung Memorial Hospital, Kaohsiung 83301, Taiwan; feymanchen@yahoo.com.tw (Y.-L.C.); shauz@cgmh.org.tw (S.-Z.C.); pather@cgmh.org.tw (S.H.); 2 Graduate Institute of Clinical Medical Sciences, College of Medicine, Chang Gung University, Taoyuan 33302, Taiwan; 3 University of Sydney, Camperdown, NSW 2006, Australia; zoid.chen@gmail.com; 4 Emergency Department, Kaohsiung Chang Gung Memorial Hospital, Kaohsiung 83301, Taiwan; gardinea1983@gmail.com; 5 Department of Neurology, Kaohsiung Chang Gung Memorial Hospital, Kaohsiung 83301, Taiwan; emily0606@cgmh.org.tw; 6 Division of Cardiology, Department of Internal Medicine, Chang Gung Memorial Hospital, Chiayi 61363, Taiwan; cmchung02@gmail.com

**Keywords:** atrial fibrillation, circadian rhythm, circadian clock genes, burden of atrial high-rate episodes, cardiac remodeling

## Abstract

Atrial fibrillation (AF) is one of the risk factors for dementia. Female sex is an inconsistent risk factor for dementia after adjusting for age in the general population, and there lacks research on its impact in developing dementia in patients with AF. This paper aims to investigate whether female sex is a risk factor for dementia in AF patients. Data of patients with newly diagnosed AF between 2001–2013 were retrieved from Taiwan’s National Health Insurance Research Database. Exclusion criteria were: patients with incomplete demographic data, age < 20 years, rheumatic heart disease, hyperthyroidism, past valvular heart surgery, and a history of dementia. Propensity score matching (PSM) between sexes was performed, including comorbidities, medications and index date stratified by age. The primary outcome was a new diagnosis of dementia at follow-up. A total of 117,517 men and 156,705 women were eligible for analysis. After 1:1 PSM, both 100,065 men and women (aged 72.5 ± 12.5 years) were included for analysis. Dementia risk varied with age in women compared with men. The difference was negligible for ≤55 years (sub distribution HR (SHR) = 0.89, 95% CI 0.73–1.07), but increased between 56–65 years (SHR = 1.13, 95% CI 1.02–1.25), 66–75 years (SHR = 1.14, 95% CI 1.09–1.20), 75–85 years (SHR = 1.11, 95% CI 1.07–1.15) and >85 years (SHR 1.10, 95% CI 1.04–1.16) for females. This study establishes that female sex increases the risk of developing dementia compared to male sex in AF patients aged >56 years. However, the impact of female sex on dementia in AF patients differs between dementia types.

## 1. Introduction

Currently, atrial fibrillation (AF) is considered to be one of the risk factors for dementia and stroke [1,2,3,4]. Previous studies have shown that women have a higher risk of stroke than men in patients with AF [5,6]. Notably, there is increased stroke severity and poorer functional outcome in women than in men after an ischemic stroke [7]. The risk of recurrent stroke and mortality is higher in women than in men after the first stroke [8]. Some of these disparities in clinical outcomes is possibly related to the older onset of ischemic stroke in women, and age could be a confounding factor in the analysis of these sex differences in ischemic strokes [9,10]. Many epidemiologic studies have also suggested that ischemic stroke is a significant risk factor for dementia, and that risk is also affected by the size and location of the presenting stroke, intercurrent medical illnesses associated with cerebral hypoxia or ischemia, and older age [11,12,13]. Previous epidemiological research has also shown that women have a higher incidence of dementia compared to men [14]. Recent studies suggest that this difference is related to the lifespan of men and women [15,16]. Current studies in Europe and America show inconsistent results after adjusting for the impact of age on dementia [17,18,19,20]. Thus, it is unclear whether female sex is a risk factor for dementia in patients with AF. The primary objective of this study is to investigate if female sex is a risk factor for developing dementia in patients with AF after adjusting for age and other associated risk factors. The secondary objective of this study is to investigate the impact of female sex on the risk of developing different types of dementia in patients with AF.

## 2. Materials and Methods

### 2.1. Data Source

Taiwan’s National Health Insurance (NHI) started in 1995 and covers 99.5% (23 million) of the residents in Taiwan. The National Health Insurance Research Database (NHIRD) stores the data for all inpatient and outpatient services, diagnoses, emergency room visits, prescriptions, examinations, operations, and expenditures, and is updated biannually. Diseases were diagnosed using the International Classification of Diseases, ninth revision, Clinical Modification (ICD-9-CM) and version 2001 codes. More detailed information about the NHIRD were outlined in a previous study [21]. This study was approved by the Institutional Review Board of Chang Gung Memorial Hospital (202002140B1). The dataset used in this study was held by the Taiwan Ministry of Health and Welfare (MOHW). Any researcher interested in accessing this dataset can submit an application form to the MOHW requesting access (Email: stcarolwu@mohw.gov.tw).

### 2.2. Study Population

**Figure 1:**
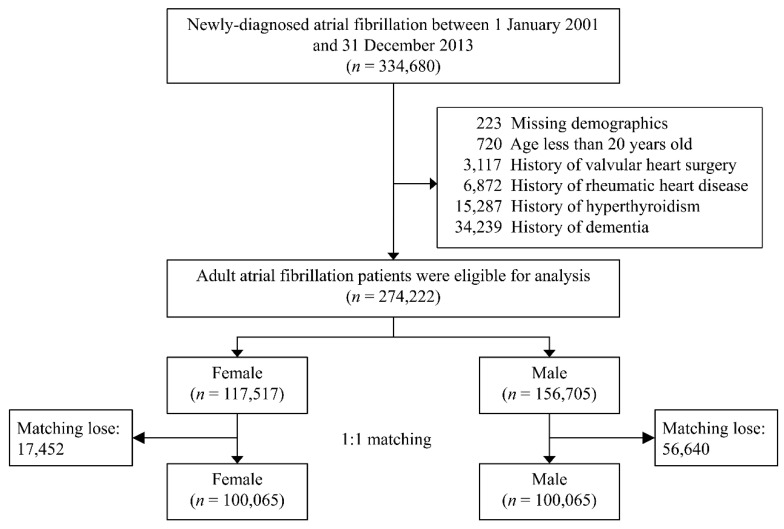
Flow diagram for the inclusion and exclusion criteria.

The electronic medical records of patients with a new diagnosis of AF (ICD-9-CM V.2001: 427.31) were retrieved from the NHIRD between the 1 January 2001 and the 31 December 2013. The date that AF was first diagnosed was assigned as the index date. The exclusion criteria were as follows: patients with incomplete demographic data (<0.1%), age < 20 years, a history of valvular heart surgery, rheumatic heart disease, and/or hyperthyroidism, and a past diagnosis of dementia. The flow diagram for the inclusion and exclusion criteria is shown in Figure 1.

### 2.3. Study Outcome

The primary outcome was a new diagnosis of dementia, which was determined by either a diagnosis of dementia on discharge, or at least two consecutive diagnoses of dementia in outpatient clinic consultations. The diagnosis of dementia was further classified into two subtypes: Alzheimer’s disease (AD) (ICD-9-CM V.2001: 331.0) and vascular dementia (ICD-9-CM V.2001: 290.4) with confirmation by positron emission tomography [PET] or single photon emission computed tomography [SPECT]. Therefore, the secondary outcome was the incidence of those 2 subtypes of dementia in the same population as the primary outcome. The diagnosis of AD was confirmed through a combination of clinical diagnosis and documented use of AD medications (i.e., donepezil, rivastigmine, memantine and galantamine). The diagnosis of vascular dementia was confirmed through clinical diagnosis of both vascular disease plus dementia and alongside PET/SPECT imaging. The definition of dementia has been outlined in previous NHIRD studies [22,23]. The diagnostic date of dementia during follow-up was assigned as the primary endpoint. Each patient was followed-up with until the date of dementia diagnosis, death, or the 31 December 2013—whichever occurred first.

### 2.4. Covariates

The study population covariates were age, socioeconomic factors (monthly income and urbanization level), comorbidities, history of cardiovascular disease, history of psychosis (depression and bipolar disorder), and medications. Comorbidities included: hypertension, diabetes mellitus, ischemic heart disease, dyslipidemia, gout, chronic obstructive pulmonary disease, peripheral arterial disease, renal function status, abnormal liver function, traumatic brain injury, and alcohol abuse. These comorbidities and any history of psychoses were determined from the discharge summary or from the two outpatient visits in the previous year. The history of cardiovascular disease included: systemic thromboembolism (not including ischemic stroke), myocardial infarction, stroke and heart failure requiring hospitalization. The history of vascular disease was identified using the discharge summaries prior to the index date, dating back to 1997. Most of these covariates and comorbidities were already validated in previous NHIRD studies [21,24,25]. Data on medication usage were retrieved on NHI claim-based data six months before the index date.

### 2.5. Ascertainment of AF and CHA_2_DS_2_-VASc Scoring

The diagnoses used to define the cohorts, comorbidities, and outcomes are listed in Table 1. The diagnosis of AF and subsequent CHA_2_DS_2_-VASc scoring in such patients (ICD-9-CM code 427.31) were determined from the medical records during previous hospitalization or during ≥2 consecutive clinic visits. The accuracy of diagnosing AF using ICD-9-CM code in the NHIRD has been validated in previous studies [26,27]. Comorbidities included in the CHA_2_DS_2_-VASc score were: hypertension, diabetes mellitus, vascular disease, stroke and heart failure, where validation have been made and appropriate medical prescriptions were utilized to increase the diagnostic accuracy [21,24,25].

### 2.6. Statistical Analysis

To adjust for any disparities in the baseline characteristics between male and female patients with AF, propensity score matching (PSM) was utilized. The patients were first allocated into 5 strata according to age (≤55, 56–65, 66–75, 76–85, and >85 years), and then PSM was applied separately for each age stratum. The 5 data sets were aggregated when conducting the overall analysis. Covariates included in the propensity score calculations are listed in Table 2, noting that the “follow-up years” refers to the duration after the index date until the study endpoints were reached. The matching was processed using the Greedy Nearest Neighbor algorithm with a caliper of 0.2 times the standard deviation of the logit of propensity score. The quality of matching was assessed using the standardized difference (STD) between sex groups after matching, where a value < 0.1 indicated negligible difference. The risk of dementia for both sexes with AF was compared in the entire matched cohort and stratified by age group using the Fine and Gray’s sub distribution hazard model, which considered all-cause death as a competing risk. The interaction of AF sex and continuous age were calculated, after which continuous age was divided into age groups. The sub distribution hazard function was used to determine the cumulative incidence of dementia events stratified by age (i.e., ≤55 years, 56–65 years, 66–75 years, 76–85 years and >85 years). Finally, forest plots comparing the risk of AD and vascular dementia between female and male patients with AF aged ≤ 55, 56–65, 66–75, 76–85 and >85 years were performed according to different CHA_2_DS_2_-VASc scores without considering the participant sex. A *p* value of <0.05 was considered statistically significant. All statistical analyses were performed using commercial software (SAS V.9.4, SAS Institute, Inc., Cary, NC, USA), including the ‘psmatch’ procedure for PSM, ‘phreg’ procedure for survival analysis, and ‘%cif’ macro for generating a cumulative incidence function through Fine and Gray’s method.

### 2.7. Patient and Public Involvement

This is a retrospective cohort study with data drawn from the NHI database. Therefore, at no stage of the research were any patients or the public involved.

## 3. Results

### 3.1. Study Population

We identified a total of 334,680 patients with a new diagnosis of AF during the 2001–2013 period in the NHIRD. After applying the exclusion criteria, 117.517 female patients with AF (aged 73.8 ± 12.4 years) and 156,705 male patients with AF (aged 70.2 ± 13.5 years) were eligible as the study population (Figure 1). Of these eligible patients, the proportion of patients aged over 75 was larger in the female population (52.9%) than in the male population (42.5%). The prevalence of hypertension and diabetes mellitus were higher in the female population while the prevalence of gout and chronic obstructive pulmonary disease were higher in the male population. Additionally, male patients had a higher prevalence of alcohol abuse and traumatic brain injury, but the female patients had higher CHA_2_DS_2_-VASc scores (left panel in Table 2). In order to balance all baseline characteristics, PSM was conducted for further comparison. Finally, equal populations of male and female patients with AF, at 100.065 patients each, were analyzed for comparison of their risk for dementia. The absolute STD values of all age groups and those of the remaining variables were all <0.1. These results suggest a balanced distribution of the baseline characteristics between male and female patients with AF in the population selected (right panel in Table 2).

### 3.2. Dementia Risk Between Sexes Through Age-Stratified Analysis Before PSM

**Figure 2:**
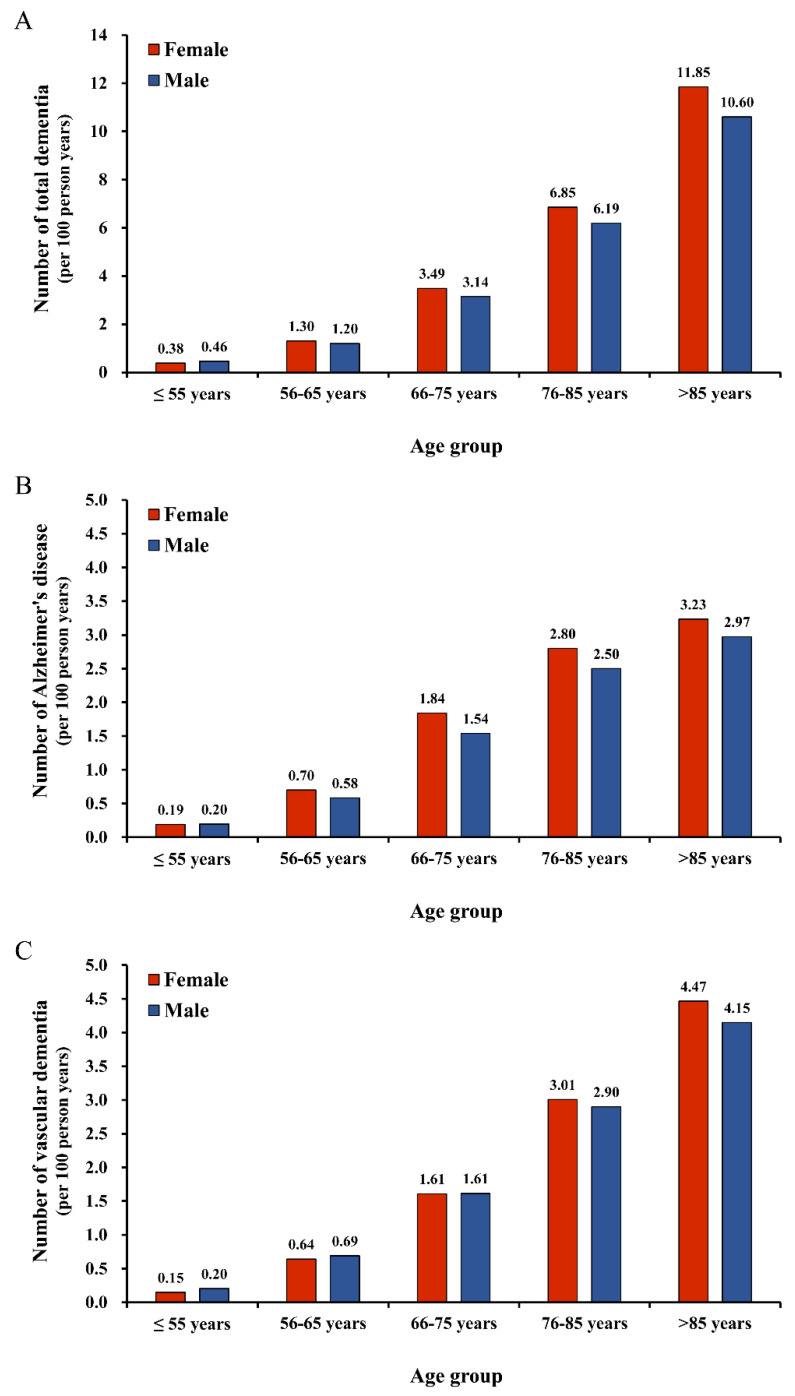
The incidence (expressed as the number of events per 100 person-years) of any dementia type (**A**), Alzheimer’s disease (**B**) and vascular dementia (**C**) in male and female patients with atrial fibrillation stratified by age distribution before propensity score matching.

Before matching, patients were stratified according to age, and this showed the incidence of total dementia events in each age group were different. Generally, with increasing age, the incidence of dementia in both groups also increased. The incidence of total dementia events was higher in women than in men in all age groups older than 55 (Figure 2A). The incidence of AD was higher in women than in men in all age groups older than 55 years old, and the incidence of vascular dementia was higher in women than in men in all age groups older than 75 (Figure 2B,C).

### 3.3. Risk of Developing Dementia between Sexes through Age-Stratified Analysis after PSM

After PSM, women have significantly higher total dementia risk compared to men in the following age groups: 56–65 (sub distribution hazard ratio [SHR] 1.13, 95% CI 1.02 to 1.25), 66–75 (SHR 1.14, 95% CI 1.09–1.20), 76–85 (SHR 1.11, 95% CI 1.07–1.15) and >85 (SHR 1.10, 95% CI 1.04–1.16) groups. The difference in total dementia risk between sexes was insignificant in the ≤55 years age group (SHR 0.89, 95% CI 0.73–1.07) (Table 3). The incidence of AD was higher in women in all age groups between 55 and 85 years old, but there was no significant difference in the incidence of vascular dementia between men and women in all age groups (Table 3).

**Figure 3:**
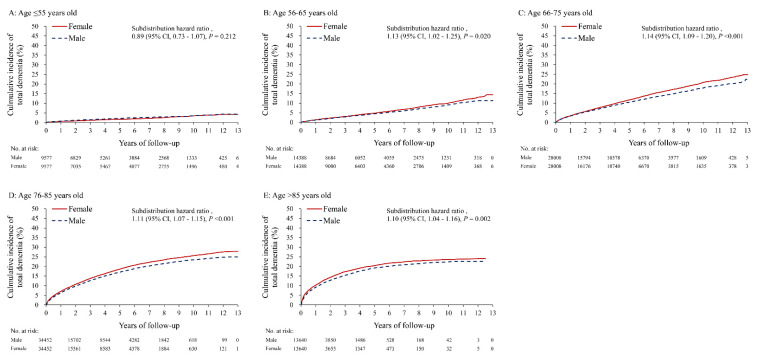
The cumulative incidence (under the Fine and Gray sub distribution model) of any dementia between male and female patients with atrial fibrillation stratified by age in the propensity score matched cohort. (**A**) age ≤ 55 years old, (**B**) age 56–65 years old, (**C**) age 66–75 years old, (**D**) age 76–85 years old, (**E**) age > 85 years old.

**Figure 4:**
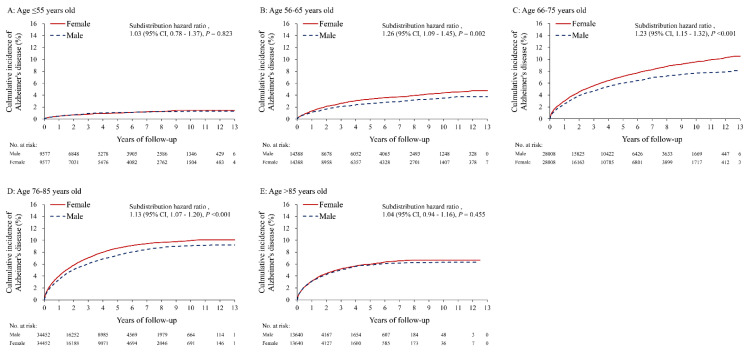
The cumulative incidence (under the Fine and Gray sub distribution model) of Alzheimer’s disease between male and female patients with atrial fibrillation stratified by age in the propensity score matched cohort. (**A**) age ≤ 55 years old, (**B**) age 56–65 years old, (**C**) age 66–75 years old, (**D**) age 76–85 years old, (**E**) age > 85 years old.

**Figure 5:**
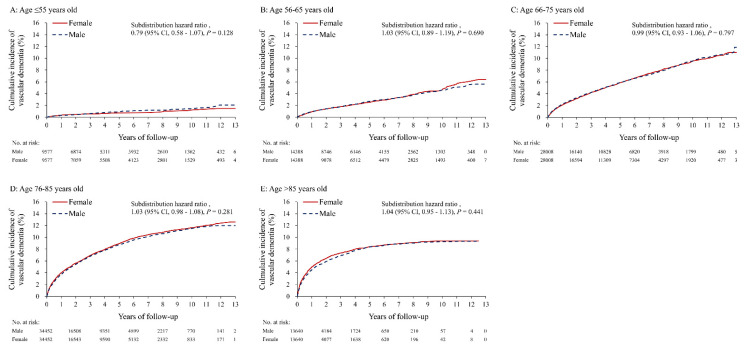
The cumulative incidence (under the Fine and Gray sub distribution model) of vascular dementia between male and female patients with atrial fibrillation stratified by age in the propensity score matched cohort. (**A**) age ≤ 55 years old, (**B**) age 56–65 years old, (**C**) age 66–75 years old, (**D**) age 76–85 years old, (**E**) age > 85 years old.

**Figure 6:**
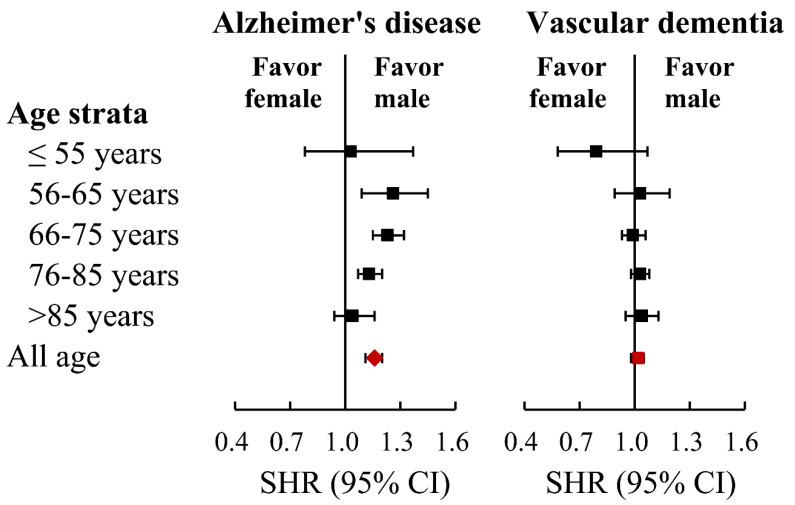
Forest plots for demonstrating the sex differences in the risk of Alzheimer’s disease and vascular dementia across different age groups in the propensity score matched cohort.

The cumulative incidence of total dementia in both sexes showed that there was no difference in the age group ≤ 55, but there was a higher incidence in women than in men in all groups older than 55 (Figure 3A–E). In contrast, the cumulative incidence of AD in both sexes showed that there was no difference between the sexes in the groups aged ≤ 55, and >85, but a higher incidence in women than in men in the age groups between 56–85 (Figure 4A–E). However, there was no difference between women and men in the cumulative incidence of vascular dementia in all five age groups (Figure 5A–E). The overall results of the differences between the sexes in their risk of AD and vascular dementia across different age groups were summarized (Figure 6).

## 4. Discussion

There were several findings of importance in this study. Firstly, the incidence of total dementia is higher in women than in men after adjusting for the following: comorbidities, socioeconomic status, medications, and also CHA_2_DS_2_-VASc score, especially in those patients who were older than 55. Secondly, the incidence of AD and vascular dementia were different between the sexes. The incidence of AD was higher in women in the age groups between 55–85, whereas the incidence of vascular dementia was the same between the sexes.

A previous study by Chen et al. showed that the risk of dementia in women with AF is comparable to men with AF, and that having AF was associated with an increased risk of dementia, after adjusting for cardiovascular risk factors using the Cox regression model [1]. However, only 2106 participants developed AF and 1157 participants developed dementia during the 20-year follow-up in the study population. Additionally, Chen et al. did not examine the impact of sex on the risk of developing dementia in a population with AF. Furthermore, the study by Chen et al. had a relatively small sample size, whereas this study analyzed more than 200,000 new-onset AF patients, with a propensity-score matched study design that demonstrated the differences between the sexes in their risk for dementia. Additionally, previous studies did not account for the different types of dementia, which were analyzed in this study [28].

AD comprises 60–70% of all dementia cases, and two thirds of AD patients are women [14]. Because women have a longer lifespan than men, age is considered the greatest risk factor for AD [16]. Although the proportion of AD is higher in women, sex differences in the incidence of AD are unclear, and vary between countries and over different time periods [29]. Previous studies have shown that there is no difference in the incidence of AD between men and women in the United States and United Kingdom [17,20]. However, other studies conducted in Sweden and France showed that the incidence of AD was higher in women than in men, especially in patients aged older than 80 [18,19]. The causes for these discrepancies in different studies are not well explained, but may suggest that sex differences in the incidence of AD may depend on the time period during which the studies were conducted and on differing geographical regions [1,30]. Most importantly, all of the previous studies were conducted in a general population rather than a population with AF. Our study evaluated the difference between sexes in the incidence of dementia in patients with a new diagnosis of AF, which showed that the incidence of AD, but not vascular dementia, was higher in women than in men, especially in patients older than 55.

Previous studies have shown that amyloid is a type of sex-related arrhythmogenic substrate which is linked to estradiol metabolism [31]. AF can cause atrial dilatation and stretch, which may cause atrial natriuretic peptide (ANP) secretion. In addition, the elevated ANP will promote amyloid formation by stimulating estrogen receptors in the presence of 17b-oestradiol [31,32]. The accumulation of amyloid may explain the higher risk for AD in women than in men with AF, and as such, may also be the mechanism to explain our findings. Several other risk factors aside from sex differences for AD have also been discussed previously, which included: smoking, alcohol abuse, education, traumatic head injury and psychiatric symptoms. This study also included these risk factors in the propensity score matching model. Further studies focusing on the mechanism of sex dependent development of AD dementia in AF patients should be conducted. Golive et al. reported that in patients who developed AF during follow-up, dementia rates increased and did not show sex-based differences in risk [33]. Prior stroke history was an independent risk factor for the development of dementia in both sexes. In addition, other previous studies also only showed that the incidence of AD, but not the incidence of vascular dementia, was higher in women than in men [18,19].

The overall frequency of vascular dementia was higher in both men and women in this study, with no difference between sexes among patients with AF. AF is a significant risk factor for ischemic stroke, and the severity of stroke in patients with AF is greater than in patients whose stroke was due to atherosclerotic carotid artery disease [34,35]. Furthermore, there are other mechanisms that may explain how AF influenced the development of vascular dementia, including cerebral hypoperfusion, vascular inflammation, cerebral small vessel disease, and the associated risk factors between AF and vascular dementia [36]. It is also worth noting that the incidence of coronary artery disease and cerebral artery disease is higher in women after menopause, and the risk is similar between women and men in old age [37,38]. This may explain why a history of ischemic stroke may be one of the most important risk factors for developing vascular dementia in AF patients during follow-up, and why there was no difference between sexes in the incidence of vascular dementia after adjusting all possible risk factors, including a history of ischemic stroke, by propensity score matching.

Several limitations are associated with epidemiologic data from the NHIRD. Firstly, the medical records of the patients in the study were retrieved from the NHIRD using the ICD-9-CM coding system, which only records a diagnosis of AF, and does not specify the type of AF diagnosed. This is important as certain types of AF may contribute more to the risk of developing dementia than other types. Further study is needed to investigate this issue. Secondly, some patients may not have been identified due to incorrect ICD-9-CM coding input by the clinicians. Thirdly, patients who had dementia with atypical symptoms may be missed due to the lack of clinical characteristics or imaging studies. Finally, this study only revealed that the female sex had a higher risk of developing dementia than the male sex in those patients with AF, especially in AD. However, further studies should be conducted to investigate the underlying mechanisms and relationship between female sex, sex hormones, AF and their role in the development of dementia.

## 5. Conclusions

This study supports the finding that the risk of dementia is higher in women than in men in patients with AF aged older than 55. However, the impact of female sex on the risk of developing dementia in patients with AF varies in different dementia types. The risk of AD is higher in women than in men in patients with AF older than 55, whereas there is no difference between women and men with AF in all age groups in their risk of developing vascular dementia.

## Figures and Tables

**Table 1:** Diagnoses used to define the cohorts, comorbidities, and outcomes.

Disease	ICD-9 CM Disease Code
Study Cohorts	
Atrial fibrillation (AF)	427.31
Excluding Diseases	
Rheumatic heart disease	394.0, 394.1, 394.2, 395, 398.9
Hyperthyroidism	242 and any anti-thyroid drugs
Main outcome	
Dementia	290.x, 294.x, 331.0
Alzheimer disease	331.0
Vascular dementia	290.4x with PET/SPECT
Comorbidities	
Hypertension	401, 402, 403, 404, 405 and any anti-hypertension drugs
Diabetes mellitus	250 and any oral hypoglycemic drugs or insulin
Ischemic heart disease	410.x~414.x
Dyslipidemia	272 and any lipid-lowing agents
Gout	274.x
Chronic obstructive pulmonary disease	491.x, 492.x, 496.x
Peripheral arterial disease (PAD)	093.0, 437.3, 440.x, 441.x, 443.x, 444.0x, 444.22, 444.8x, 447.1, 447.8x, 447.9x, 557.1, 557.9, V43.4
Chronic kidney disease	580.x~589.x, 403.x~404.x, 016.0, 095.4, 236.9 250.4, 274.1, 442.1, 447.3, 440.1, 572.4, 642.1, 646.2, 753.1, 283.11, 403.01, 404.02, 446.21
Dialysis	585
Abnormal liver function	070, 456.0~456.2, 570, 571, 572.2~572.8, 573, V42.7
Traumatic brain injury	800.x~804.x, 850.x~854.x
Alcohol abuse	V113, 291.x, 305.0x, 357.5, 425.5, 303.x, 571.0, 571.1, 571.2, 571.3, 980.0
Systemic thromboembolism	415.1x, 444.22, 444.81, 444.21, 362.30, 362.34, 444.89, 557.0, 444.9x
Ischemic stroke	433.x~437.x
Myocardial infarction	410.x, 412.x
Stroke	430.x~437.x
Heart failure	428.x
Depression	296.2x, 296.3x
Bipolar	296.x

ICD-9-CM, International Classification of Diseases, Ninth Revision, Clinical Modification; PET, positron emission tomography; SPECT, single photon emission computed tomography.

**Table 2:** Baseline characteristics of the study patients before and after propensity score matching.

	Before Matching				After Matching		
Variables	Total (*n* = 274,222)	Female (*n* = 117,517)	Male (*n* = 156,705)	STD	Female (*n* = 100,065)	Male (*n* = 100,065)	STD
Age (years)	71.8 ± 13.2	73.8 ± 12.4	70.2 ± 13.5	0.28	72.5 ± 12.5	72.5 ± 12.5	0.00
Age group							
≤55 years	31,738 (11.6)	9641 (8.2)	22,097 (14.1)	−0.19	9577 (9.6)	9577 (9.6)	0.00
56–65 years	41,497 (15.1)	14,788 (12.6)	26,709 (17.0)	−0.13	14,388 (14.4)	14,388 (14.4)	0.00
66–75 years	72,265 (26.4)	30,993 (26.4)	41,272 (26.3)	0.00	28,008 (28.0)	28,008 (28.0)	0.00
76–85 years	91,424 (33.3)	41,927 (35.7)	49,497 (31.6)	0.09	34,452 (34.4)	34,452 (34.4)	0.00
>85 years	37,298 (13.6)	20,168 (17.2)	17,130 (10.9)	0.18	13,640 (13.6)	13,640 (13.6)	0.00
Monthly income, USD							
≤600	166,993 (60.9)	72,897 (62.0)	94,096 (60.0)	0.04	63,393 (63.4)	63,480 (63.4)	0.00
601–800	86,118 (31.4)	39,507 (33.6)	46,611 (29.7)	0.08	31,566 (31.5)	31,480 (31.5)	0.00
>800	21,111 (7.7)	5113 (4.4)	15,998 (10.2)	−0.23	5106 (5.1)	5105 (5.1)	0.00
Urbanization level							
Low	39,115 (14.3)	18,071 (15.4)	21,044 (13.4)	0.06	14,249 (14.2)	14,304 (14.3)	0.00
Moderate	89,523 (32.6)	38,516 (32.8)	51,007 (32.5)	0.00	32,521 (32.5)	32,532 (32.5)	0.00
High	80,059 (29.2)	33,046 (28.1)	47,013 (30.0)	−0.04	29,279 (29.3)	29,196 (29.2)	0.00
Very high	65,525 (23.9)	27,884 (23.7)	37,641 (24.0)	−0.01	24,016 (24.0)	24,033 (24.0)	0.00
Comorbidities							
Hypertension	173,413 (63.2)	79,158 (67.4)	94,255 (60.1)	0.15	64,159 (64.1)	64,092 (64.1)	0.00
Diabetes mellitus	69,355 (25.3)	33,120 (28.2)	36,235 (23.1)	0.12	25,859 (25.8)	25,817 (25.8)	0.00
Ischemic heart disease	101,722 (37.1)	42,706 (36.3)	59,016 (37.7)	−0.03	36,186 (36.2)	36,202 (36.2)	0.00
Dyslipidemia	49,457 (18.0)	22,705 (19.3)	26,752 (17.1)	0.06	17,867 (17.9)	17,710 (17.7)	0.00
Gout	28,848 (10.5)	7359 (6.3)	21,489 (13.7)	−0.25	7253 (7.2)	7236 (7.2)	0.00
COPD	51,732 (18.9)	15,261 (13.0)	36,471 (23.3)	−0.27	15,048 (15.0)	14,909 (14.9)	0.00
Peripheral arterial disease	13,311 (4.9)	5689 (4.8)	7622 (4.9)	0.00	4810 (4.8)	4790 (4.8)	0.00
Renal function status							
Non-CKD	235,735 (86.0)	101,229 (86.1)	134,506 (85.8)	0.01	86,351 (86.3)	86,316 (86.3)	0.00
CKD without dialysis	30,624 (11.2)	12,141 (10.3)	18,483 (11.8)	−0.05	10,640 (10.6)	10,745 (10.7)	0.00
CKD with dialysis	7863 (2.9)	4147 (3.5)	3716 (2.4)	0.07	3074 (3.1)	3004 (3.0)	0.00
Abnormal liver function	30,337 (11.1)	11,303 (9.6)	19,034 (12.1)	−0.08	10,107 (10.1)	10,079 (10.1)	0.00
Traumatic brain injury	5531 (2.0)	2111 (1.8)	3420 (2.2)	−0.03	1920 (1.9)	1934 (1.9)	0.00
Alcohol abuse	2767 (1.0)	223 (0.2)	2544 (1.6)	−0.15	223 (0.2)	233 (0.2)	0.00
History of events							
Systemic thromboembolism (excluding ischemic stroke)	4708 (1.7)	2093 (1.8)	2615 (1.7)	0.01	1694 (1.7)	1656 (1.7)	0.00
Myocardial infarction	11,916 (4.3)	3796 (3.2)	8120 (5.2)	−0.10	3559 (3.6)	3555 (3.6)	0.00
Stroke	37,376 (13.6)	16,501 (14.0)	20,875 (13.3)	0.02	13,639 (13.6)	13,613 (13.6)	0.00
Heart failure	34,697 (12.7)	16,456 (14.0)	18,241 (11.6)	0.07	12,207 (12.2)	12,164 (12.2)	0.00
History of psychosis							
Depression	1896 (0.7)	1010 (0.9)	886 (0.6)	0.03	708 (0.7)	670 (0.7)	0.00
Bipolar	2889 (1.1)	1483 (1.3)	1406 (0.9)	0.04	1081 (1.1)	1022 (1.0)	0.01
Medications							
Anticoagulant	62,373 (22.7)	26,406 (22.5)	35,967 (23.0)	−0.01	22,955 (22.9)	23,032 (23.0)	0.00
Antiplatelet	94,571 (34.5)	38,573 (32.8)	55,998 (35.7)	−0.06	33,646 (33.6)	33,692 (33.7)	0.00
ACEi/ARB	113,312 (41.3)	50,462 (42.9)	62,850 (40.1)	0.06	41,141 (41.1)	41,008 (41.0)	0.00
Dihydropyridine CCB	68,488 (25.0)	32,585 (27.7)	35,903 (22.9)	0.11	25,692 (25.7)	25,564 (25.5)	0.00
Non-dihydropyridine CCB	46,929 (17.1)	21,102 (18.0)	25,827 (16.5)	0.04	17,314 (17.3)	17,198 (17.2)	0.00
ß-blockers	93,261 (34.0)	42,680 (36.3)	50,581 (32.3)	0.09	34,887 (34.9)	34,622 (34.6)	0.01
Statins	32,312 (11.8)	14,409 (12.3)	17,903 (11.4)	0.03	11,696 (11.7)	11,547 (11.5)	0.00
DPP4 inhibitors	5604 (2.0)	2560 (2.2)	3044 (1.9)	0.02	2053 (2.1)	2093 (2.1)	0.00
Biguanides	26,651 (9.7)	12,758 (10.9)	13,893 (8.9)	0.07	10,094 (10.1)	10,014 (10.0)	0.00
Sulfonylurea	29,146 (10.6)	13,916 (11.8)	15,230 (9.7)	0.07	10,967 (11.0)	10,971 (11.0)	0.00
Thiazolidinedione	3769 (1.4)	1808 (1.5)	1961 (1.3)	0.02	1488 (1.5)	1454 (1.5)	0.00
Insulin	8563 (3.1)	4471 (3.8)	4092 (2.6)	0.07	3237 (3.2)	3160 (3.2)	0.00
CHA_2_DS_2_-VASc score *	3.3 ± 1.9	4.1 ± 1.7	2.8 ± 1.7	0.79	4.0 ± 1.7	2.9 ± 1.7	0.58
CHA_2_DS_2_-VASc score group *							
0	16,156 (5.9)	0 (0.0)	16,156 (10.3)	−0.48	0 (0.0)	8718 (8.7)	−0.44
1	31,510 (11.5)	9137 (7.8)	22,373 (14.3)	−0.21	9064 (9.1)	11,465 (11.5)	−0.08
2	44,829 (16.3)	11,474 (9.8)	33,355 (21.3)	−0.32	11,324 (11.3)	20,077 (20.1)	−0.24
3	56,571 (20.6)	21,578 (18.4)	34,993 (22.3)	−0.10	19,981 (20.0)	24,109 (24.1)	−0.10
4	54,259 (19.8)	28,785 (24.5)	25,474 (16.3)	0.21	23,928 (23.9)	18,193 (18.2)	0.14
5	36,594 (13.3)	22,812 (19.4)	13,782 (8.8)	0.31	17,865 (17.9)	9863 (9.9)	0.23
6	20,074 (7.3)	12,976 (11.0)	7098 (4.5)	0.24	9880 (9.9)	5082 (5.1)	0.18
7–9	14,229 (5.2)	10,755 (9.2)	3474 (2.2)	0.30	8023 (8.0)	2558 (2.6)	0.25
Follow-up years *	3.4 ± 3.3	3.5 ± 3.3	3.4 ± 3.3	0.02	3.5 ± 3.4	3.4 ± 3.3	0.03
Propensity score	0.429 ± 0.159	0.488 ± 0.145	0.384 ± 0.155	0.69	0.457 ± 0.132	0.456 ± 0.132	0.01

STD, standardized difference; USD, United States Dollar; COPD, chronic obstructive pulmonary disease; CKD, chronic kidney disease; ACEi, angiotensin converting enzyme inhibitors; ARB, angiotensin II receptor blockers; CCB, calcium channel blockers; DPP4, Dipeptidyl peptidase 4; * Indicates this variable was not included in the propensity score; Data were expressed as mean ± standard deviation or frequency (percentage).

**Table 3:** The risk of dementia between sexes after propensity score matching.

Type of Dementia/Age	Female (*n* = 100,065)			Male (*n* = 100,065)			Female vs. Male	
	Patient	Event (%)	ID (95% CI)	Patient	Event (%)	ID (95% CI)	SHR (95% CI)	*p*-Value
Any dementia								
≤55 years	9577	198 (2.1)	0.39 (0.33–0.44)	9577	223 (2.3)	0.45 (0.39–0.51)	0.89 (0.73–1.07)	0.212
56–65 years	14,388	780 (5.4)	1.28 (1.19–1.37)	14,388	696 (4.8)	1.20 (1.11–1.29)	1.13 (1.02–1.25)	0.020
66–75 years	28,008	3478 (12.4)	3.40 (3.29–3.52)	28,008	3087 (11.0)	3.10 (2.99–3.21)	1.14 (1.09–1.20)	<0.001
76–85 years	34,452	6022 (17.5)	6.78 (6.61–6.95)	34,452	5500 (16.0)	6.18 (6.02–6.34)	1.11 (1.07–1.15)	<0.001
>85 years	13,640	2389 (17.5)	11.94 (11.46–12.42)	13,640	2211 (16.2)	10.40 (9.97–10.83)	1.10 (1.04–1.16)	0.002
Total	100,065	12,867 (12.9)	3.98 (3.91–4.05)	100,065	11,717 (11.7)	3.70 (3.63–3.77)	1.11 (1.08–1.14)	<0.001
Alzheimer’s disease								
≤55 years	9577	98 (1.0)	0.19 (0.15–0.23)	9577	95 (1.0)	0.19 (0.15–0.23)	1.03 (0.78–1.37)	0.823
56–65 years	14,388	415 (2.9)	0.69 (0.62–0.75)	14,388	333 (2.3)	0.58 (0.51–0.64)	1.26 (1.09–1.45)	0.002
66–75 years	28,008	1856 (6.6)	1.80 (1.72–1.89)	28,008	1521 (5.4)	1.52 (1.44–1.60)	1.23 (1.15–1.32)	<0.001
76–85 years	34,452	2593 (7.5)	2.80 (2.70–2.91)	34,452	2309 (6.7)	2.50 (2.40–2.60)	1.13 (1.07–1.20)	<0.001
>85 years	13,640	693 (5.1)	3.13 (2.89–3.36)	13,640	667 (4.9)	2.93 (2.71–3.15)	1.04 (0.94–1.16)	0.455
Total	100,065	5655 (5.7)	1.72 (1.67–1.76)	100,065	4925 (4.9)	1.53 (1.48–1.57)	1.16 (1.11–1.20)	<0.001
Vascular dementia								
≤55 years	9577	76 (0.8)	0.15 (0.11–0.18)	9577	96 (1.0)	0.19 (0.15–0.23)	0.79 (0.58–1.07)	0.128
56–65 years	14,388	391 (2.7)	0.63 (0.57–0.69)	14,388	381 (2.7)	0.65 (0.58–0.71)	1.03 (0.89–1.19)	0.690
66–75 years	28,008	1674 (6.0)	1.56 (1.48–1.63)	28,008	1691 (6.0)	1.64 (1.56–1.72)	0.99 (0.93–1.06)	0.797
76–85 years	34,452	2854 (8.3)	2.97 (2.86–3.08)	34,452	2785 (8.1)	2.93 (2.82–3.04)	1.03 (0.98–1.08)	0.281
>85 years	13,640	988 (7.2)	4.45 (4.17–4.73)	13,640	960 (7.0)	4.16 (3.90–4.43)	1.04 (0.95–1.13)	0.441
Total	100,065	5983 (6.0)	1.76 (1.72–1.81)	100,065	5913 (5.9)	1.79 (1.75–1.84)	1.02 (0.98–1.05)	0.429

ID, incidence density; SHR, sub distribution hazard ratio; CI, confidence interval.

## Data Availability

The dataset used in this study was held by the Taiwan Ministry of Health and Welfare (MOHW). Any researcher interested in accessing this dataset can submit an application form to the MOHW requesting access (Email: stcarolwu@mohw.gov.tw).
